# Effects of cinnamon nanoparticles produced with ultrasonic waves on growth performance, antioxidant status, and hemato-biochemical metabolites of broiler chickens

**DOI:** 10.1016/j.psj.2025.105615

**Published:** 2025-07-28

**Authors:** Mokhtar Fathi, Kianoosh Zarrinkavyani, Zahra Biranvand, Ali Hashem Mohammad

**Affiliations:** a Department of Animal Science, Payame Noor University, Tehran, Iran; b Department of Animal Science, Faculty of Agriculture, Ilam University, Ilam, Iran; c Researcher, Department of Animal Science, Ilam Agricultural and Natural Resources Research and Education Center, AREEO, Ilam, Iran

**Keywords:** Antioxidant, Broiler, Biochemical metabolite, Cinnamon nanoparticles, Performance

## Abstract

A comprehensive experiment was carried out to evaluate the impact of cinnamon nanoparticles (CNPs) on growth performance and hemato-biochemical parameters in broiler chickens. A total of 500 one-day-old Ross 308 chicks were randomly distributed into five dietary treatment groups in a completely randomized design, with each treatment consisting of five replicates containing 20 birds per replicate. The experimental treatments included: A control group receiving a basal diet without supplementation, and four groups supplemented with 150, 300, 450, or 600 mg of CNPs per kg of feed. CNPs were synthesized using an ultrasonic-assisted method. Results demonstrated that dietary inclusion of CNPs at 300–600 mg/kg significantly enhanced average daily weight gain, reduced feed conversion ratio, and decreased mortality (P < 0.05), and with the most pronounced benefits observed at the 450 mg/kg level. Supplementation with CNPs also improved the antioxidant defense system, as indicated by elevated total antioxidant capacity, superoxide dismutase (SOD), and glutathione peroxidase (GPx) activities, along with a marked reduction in serum malondialdehyde (MDA) levels (P < 0.05). These effects were most prominent at 450 mg/kg. Furthermore, supplementation with 450 and 600 mg/kg of cinnamon nanoparticles (CNPs) significantly decreased red blood cell (RBC) count, hemoglobin concentration, hematocrit values, heterophil percentage, and cortisol levels, while significantly increasing lymphocyte percentage (P < 0.05).. Additionally, dietary CNPs led to a reduction in serum concentrations of cholesterol, triglycerides, low-density lipoprotein (LDL), uric acid, creatinine, and aspartate aminotransferase (AST). In conclusion, dietary inclusion of 450 mg/kg CNPs was found to optimally improve growth performance, enhance antioxidant capacity, and positively modulate hematological and biochemical profiles in broiler chickens.

## Introduction

Broiler chickens grow rapidly and convert feed efficiently (Yu et al., 2025). However, their fast growth raises oxygen demand, which can cause hypoxemia and increased production of reactive oxygen species (ROS), leading to oxidative stress (Rahmani et al., 2018). Oxidative stress negatively impacts broiler health and performance, with malondialdehyde (MDA) as a key biomarker and enzymes like SOD, CAT, and GSH-Px indicating antioxidant defense efficiency (Fathi et al., 2022; Surai, 2016). Plant-based antioxidants can reduce oxidative damage by modulating these markers. Although many herbal extracts are studied, cinnamon—known for its bioactive compounds like cinnamaldehyde and polyphenols with strong antioxidant properties—remains less explored, especially in nanoparticle form (Abdel-Tawwab et al., 2018; Kwon et al., 2009; Luczaj et al., 2009). Cinnamon's antioxidant, anti-inflammatory, and antimicrobial effects support its potential as a natural feed additive to improve broiler growth and oxidative stability (Lee et al., 2005; Abdel-Tawwab et al., 2018).

However, the limited bioavailability of herbal extracts, caused by poor intestinal absorption and rapid metabolism, restricts their therapeutic potential**.** To overcome this limitation, advanced delivery systems such as liposomes, phospholipid complexes, microemulsions, polymeric micelles, and nanoparticles have been developed (Bisht & Maitra, 2009; Liu et al., 2016). Among these, nanoparticle-based formulations have shown particular promise in enhancing oral absorption and bioefficacy.

Specifically, cinnamon nanoparticles (CNPs) have been proposed to improve cinnamon's bioavailability by enhancing its solubility and intestinal permeability (Abdel-Tawwab et al., 2018). Previous studies demonstrate that nanoparticle formulations can significantly increase the bioavailability and biological half-life of phytochemicals, as shown for curcumin in both rodent and broiler models (Ma et al., 2007; Rahmani et al., 2018).

Compared to its natural form, cinnamon processed into nanoparticles (CNPs) offers several significant advantages. The natural form of cinnamon contains a range of bioactive compounds, including cinnamaldehyde, polyphenols, and flavonoids; however, its therapeutic potential is often limited by poor aqueous solubility, low intestinal permeability, rapid metabolism, and fast systemic elimination (Abdel-Tawwab et al., 2018). Nanoparticulation addresses these challenges by reducing particle size, thereby increasing surface area, improving solubility, and enhancing absorption across the gastrointestinal tract (Rahmani et al., 2018). Furthermore, CNPs may offer controlled release properties and improved cellular uptake, resulting in greater bioavailability and prolonged biological activity (Abdel-Tawwab et al., 2018). These enhancements make nanoparticle-based cinnamon formulations a promising alternative to traditional forms, particularly for use in animal nutrition where consistent and efficient delivery of active compounds is critical. Based on these considerations, we hypothesize that dietary supplementation with cnps can improve feed efficiency, enhance antioxidant defenses, and promote physiological health and performance in broiler chickens. Therefore, this study was designed to evaluate the effects of dietary CNP supplementation on growth performance, antioxidant enzyme activity, and hematological profiles in broilers.

## Materials and methods

### Ethics statement

All animal-related experimental protocols were reviewed and approved by the Animal Ethics and Welfare Committee of the Department of Animal Science at Payame Noor University (Approval Code: IR.PNU.REC.1404.148). The entire study was conducted in strict compliance with the national ethical guidelines for the care and use of laboratory animals, as issued by the Iranian Ministry of Science, Research, and Technology. All necessary measures were undertaken to minimize animal suffering and to ensure the use of the minimum number of animals required to achieve statistical validity.

### Experimental birds and diet

A total of 500 one-day-old Ross 308 broiler chicks were randomly assigned to five dietary treatment groups in a completely randomized design. Each treatment consisted of five replicates, with 20 chicks allocated per replicate. The five experimental groups included: Control group receiving a standard basal diet, and groups supplemented with 150, 300, 450, and 600 mg of cinnamon nanoparticles (CNPs) per kilogram of feed, respectively. The doses of cinnamon nanoparticles used in this study were selected based on previous research demonstrating effective antioxidant and growth-promoting effects in broilers (Abdel-Tawwab et al., 2018; Rahmani et al., 2018). These doses aim to balance efficacy and safety to optimize broiler performance and health.

A continuous lighting regimen of 23 hours light and 1 hour dark was maintained throughout the entire experimental period. Birds were housed in floor pens equipped with circular feeders and drinkers, each measuring 150 × 200 cm per replicate. All broilers were vaccinated in accordance with standard protocols for Newcastle disease and other common infectious poultry diseases. The diets were formulated based on corn and soybean meal to meet the nutritional requirements recommended for Ross 308 broilers. Three feeding phases were implemented: a starter diet (days 1–10), a grower diet (days 11–24), and a finisher diet (days 25–42), as outlined in Table 1. Feed and fresh water were provided ad libitum throughout the trial.

**Table 1:** The ingredients and composition of the basal diet.

	Starter (0 to10 days)	Grower (11 to 24 days)	Finisher (25 to 42 days)
Ingredients (%)			
Mize, 8% CP	47.53	51.63	57.56
Soybean meal, 44%CP	42.35	37.99	32.35
Soybean oil, 9000 kcal/kg	5.54	6.24	6.29
Limestone, 38% Ca	1.20	1.12	1.05
Di-calcium phosphate, 21%Ca	1.79	1.56	1.34
Vitamin premix^b^	0.25	0.25	0.25
Mineral premix^c^	0.25	0.25	0.25
NaCl	0.40	0.40	0.40
DL-Methionine, 99%	0.37	0.32	0.28
Lysine, 78%	0.28	0.22	0.22
Threonine, 98.5%	0.05	0.02	0.00
Calculated values ^d^			
Metabolizable energy, kCal/kg	2990	3082	3218
Crude protein, %	23	21.3	19.3
Calcium (Ca), %	0.96	0.87	0.79
Available phosphorus, %	0.456	0.409	0.361
Sodium (Na), %	0.16	0.16	0.16
Methionine, %	0.71	0.64	0.58
Methionine‏cysteine, %	1.07	0.89	0.89
Lysine, %	1.46	1.30	1.17
Arginine, %	1.56	1.45	1.30
Threonine, %	0.96	0.87	0.78
Tryptophan, %	0.35	0.32	0.29

b Vitamin concentrations per kilogram of diet: 4,500 IU vitamin A; 4000 IU vitamin D3; 3000 IU vitamin E; vitamin K3, 2 mg; thiamin, 2 mg; riboflavin, 6.00 mg; biotin, 0.1 mg; cobalamin, 0.015 mg; pyroxidine, 3 mg; niacin, 11.00 mg; d-pantothenic acid, 25.0; menadione sodium bisulphate, 1.10; folic acid, 1.02; choline chloride, 250 mg; nicotinamide, 5 mg;

C Mineral concentrations per kilogram of diet:calcium pantothenate, 25 mg; Fe (from ferrous sulphate), 35 mg; Cu (from copper sulphate), 3.5 mg; Mn (from manganese sulphate), 60 mg; Zn (from zinc sulphate), 35 mg; I (from calcium iodate), 0.6 mg; Se (from sodium selenite), 0.3 mg.

### Cinnamon nanoparticles (CNPs) preparation

**Fig. 1:**
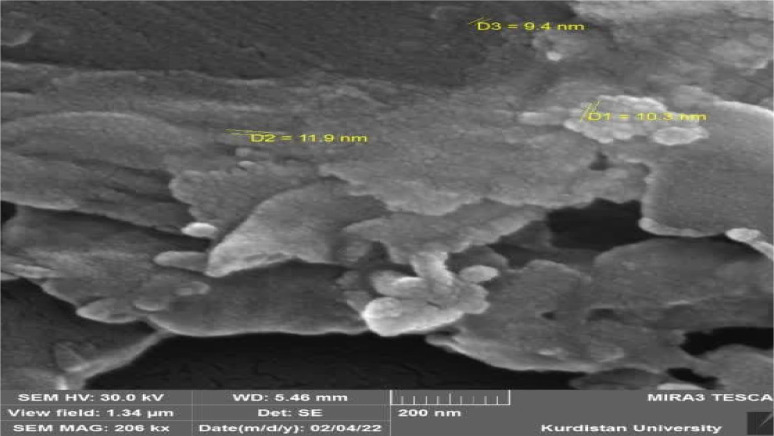
Electron microscope image of a cinnamon particle at different magnifications.

The ultrasonic method was used to synthesize cinnamon nanoparticles. This method is based on the use of ultrasonic energy. The ultrasonic method is based on a phenomenon called cavitation, which involves the creation of a series of bubbles due to the application of sonic waves in a solution. The process that occurs due to the application of ultrasonic waves in a solution includes the formation, growth and explosion of bubbles in the solution. For this purpose, different proportions of dry cinnamon extract in ethanol and at temperatures of 25 and 50°C were placed in an ultrasonic device (BANDELIN SONOPLUS model, Faraz Teb Tajhiz Company, Iran) under a power of 300 W for 60 minutes. The best performance and the highest dispersion were observed in dissolving 5 grams of cinnamon powder in 400 cc of ethanol at 25°C. In the remaining cases, the cinnamon powder appeared lumpy and sticky. Finally, to confirm the synthesis of the desired nanoparticles, scanning electron microscopy (SEM) was used. The electron microscope images showed the spherical shape of the nanoparticles and a size below 50 nm (Fig. 1). The results of counting the nanoparticles compared to the total particles in the solution showed that the percentage of nanoparticles to the total particles was 68%. The dry cinnamon extract (cinnamon) with the trade name used in this study was a product of Adonis Gol Daru Company (Tehran, Iran), which was packaged in a 1-kilogram bag of dark brown powder with a purity of 95% and containing 10.31% of the active ingredient cinnamaldehyde. The ethanol used was also obtained from Pars Medico Company (Tehran, Iran), with a purity of 99%.

### Broiler chicken's growth performance analysis

Body weights of individual broiler chickens were recorded on days 14 and 42 of the experiment. At the end of the trial, body weight gain (BWG) and feed intake (FI) were measured, and the feed conversion ratio (FCR) was calculated for the 28-day period. Daily health inspections were performed, and all mortalities were recorded along with their respective dates. FCR was determined by dividing total feed intake by body weight gain (FCR = FI/BWG), with adjustments made to account for the body weight of deceased birds, in accordance with the method described by Bahrampour et al. (2021).

In this study, performance measurements were conducted only at the end of the experimental period (day 42). Due to time and resource constraints, data collection at intermediate ages (e.g., days 14 and 28) was not feasible. However, focusing on end-point results provides a comprehensive view of the overall effects of dietary supplementation throughout the growth period.

### Blood sampling for hematological and biochemistry indices

On day 42 of age, ten birds from each treatment group (two birds per replicate) were randomly selected and slaughtered following an overnight fasting period. Birds were euthanized via exsanguination, and blood samples were collected immediately. Serum was separated by centrifugation at 3,500 rpm for 12 minutes at 4°C and stored at −20°C for subsequent analyses of biochemical and oxidative stress parameters (Fathi et al., 2022). Hematological indices were measured using an automated hematology analyzer (Sysmex KX-21N, Japan). Differential leukocyte counts were performed manually according to the method described by Rasha et al. (2017). For further biochemical analysis, serum samples were again centrifuged at 2,500 × g for 10 minutes at room temperature and preserved at −20°C (Fathi et al., 2022).

Serum concentrations of cortisol, creatinine, urea, uric acid, alanine aminotransferase (ALT), aspartate aminotransferase (AST), total cholesterol (TC), triglycerides (TG), low-density lipoprotein (LDL), and high-density lipoprotein (HDL) were determined using an automated biochemistry analyzer (Alcyon 300, Abbott, USA) with commercially available diagnostic kits (Pars Azmoon, Tehran, Iran).

Glutathione peroxidase (GSH-Px) activity was measured using the Ransel kit (RS-504, Randox Laboratories, Crumlin, UK). Catalase (CAT) and total superoxide dismutase (T-SOD) activities were determined using the RANSOD kit (SD-125, Randox Laboratories, UK). All enzyme assays were conducted according to the manufacturers’ protocols using the aforementioned autoanalyzer. Malondialdehyde (MDA) concentration, an indicator of lipid peroxidation and oxidative stress, was quantified in serum following the method described by Fathi et al. (2022). MDA represents the end product of lipid peroxidation triggered by oxidative stress and was used as a biomarker to evaluate oxidative damage.

### Statistical analysis

Statistical analyses were performed using the General Linear Model (GLM) procedure in SAS software (version 11; SPSS Inc., Chicago, IL, USA) to assess the effect of treatments on the measured parameters. The applied model was structured as follows: Yij = µ + Ti + eij. Where Y ij = observed value; µ = overall mean; Ti = treatment); and eij = random error. The differences between means were compared by Tukey’s test at 5% of probability. The SEM and mean values were reported. The linear and quadratic effects of CNPs were detected by orthogonal polynomials. The differences between treatments are considered significant at P < 0.05. Mortality rate was transformed to log_10_ to normalize the data distribution before running the statistical analysis.

## Result

### Growth performance and mortality

The effects of different experimental treatments on the growth performance and mortality of broiler chickens are presented in Table 2. The results indicated that dietary supplementation with 450 and 600 mg of CNPs significantly increased feed intake (FI) throughout the experimental period compared to the other treatments (P < 0.05), with the highest FI observed in the group receiving 600 mg of CNPs. Moreover, birds supplemented with 300, 450, and 600 mg/kg of CNPs showed significantly improved body weight gain (BWG) at 42 days of age compared to the control group (P < 0.05). Notably, birds receiving 300 and 450 mg/kg CNPs exhibited improved feed conversion ratios (FCR), reflecting better feed efficiency compared to the control group.

**Table 2:** Growth performance of broiler chicks as affected by dietary supplementation of cinnamon nanoparticles (CNPs) at 6 wk of age.

Items	CNPs level (mg/kg diet)					SEM	P-Value	
	0	150	300	450	600		Linear	Quadratic
Feed Intake [g]	3937.0^b^	3859.5^b^	3867.6^b^	3742.2^c^	4352.4^a^	31.66	< 0.010	< 0.010
Body weight gain [g]	2540.0^c^	2490.0^c^	2930.0^a^	2970^a^	2790^b^	75.43	< 0.001	< 0.010
Body weight gain [g/d]	60.47^c^	59.28^c^	69.76^a^	70.71^a^	66.42^b^	1.03	< 0.010	< 0.010
Feed conversion ratio [g feed/g gain]	1.55^a^	1.55^a^	1.32^b^	1.26^c^	1.56^a^	0.027	< 0.030	< 0.010
Mortality (%)	4.00^a^	0.02^b^	0.02^b^	0.00^c^	0.02^b^	0.03	< 0.010	< 0.010

^a, b, c^ Mean values in the same row with different superscript letters were significantly. (n = 10).

Administration of CNPs at all levels significantly reduced mortality during the 1–42 day experimental period. Moreover, the lowest mortality rates were observed in the groups receiving 450 and 600 mg of CNPs (P<0.05). The most favorable overall growth performance and lowest mortality was recorded in the group receiving 450 mg of CNPs (P<0.05).

### **Antioxidant***indices*

Data in Table 3 show that CNPs supplementation significantly affected antioxidant capacity of serum in broiler chickens (P<0.05). The results showed that while CNPs supplementation had no significant effect on serum catalase (CAT) levels, all levels of CNPs significantly increased glutathione peroxidase (GSH-Px) activity and reduced serum malondialdehyde (MDA) levels (P<0.05). The highest GSH-Px activity was observed in the group supplemented with 300 mg of CNPs, whereas the lowest serum MDA level was recorded in the group receiving 450 mg of CNPs (P<0.05). Additionally, the highest superoxide dismutase (SOD) activity was observed in the group receiving 450 mg of CNPs (P<0.05).

**Table 3:** Antioxidant status in serum of broiler chicks as affected by dietary supplementation of cinnamon nanoparticles (CNPs) at 6 week of age.

Items	CNPs level (mg/kg diet)					SEM	P-Value	
	0	150	300	450	600		Linear	Quadratic
GSH-Px (U/mL)	1809.12^d^	1871.45^bc^	1896.56^ab^	1885.14^bc^	1921.40^a^	9.55	< 0.01	0.41
T-SOD (U/mL)	281.11^bc^	290.12^bc^	303.12^b^	311.62^b^	380.65^a^	6.68	< 0.01	0.236
CAT (U/mL)	91.16	95.055	94.77	99.25	96.55	3.02	0. 201	0.19
MDA (n mol/mL)	19.82^a^	15.20^b^	13.12^bc^	12.65^bc^	11.22^c^	1.09	< 0.02	0.391

^a, b, c^ Mean values in the same row with different superscript letters were significantly. (n = 10).

GSH-Px, glutathione peroxidase; T-SOD, Total superoxide dismutase; CAT, catalase; MDA, malondialdehyde.

### **Hematological***indices*

The effects of dietary supplementation with varying levels of cinnamon nanoparticles (CNPs) on hematological parameters of broiler chickens are summarized in Table 4. Supplementation with higher doses of CNPs (450 and 600 mg/kg) resulted in a significant reduction in red blood cell count, hematocrit, and hemoglobin levels compared to other groups (P<0.05), which may indicate a potential adverse impact on oxygen-carrying capacity. Conversely, supplementation at 300 mg/kg and above significantly increased lymphocyte percentages and decreased heterophil percentages, along with a reduction in blood cortisol levels (P<0.05), suggesting a possible modulation of immune response and stress reduction. Total white blood cell counts remained unaffected by the different CNP doses (P>0.05). Overall, while some hematological changes may raise concerns, the most beneficial effects were noted at 300 and 450 mg/kg, indicating a dose-dependent response that requires careful consideration of both positive and negative outcomes.

**Table 4:** Hematological values of broiler chicks as affected by dietary supplementation of cinnamon nanoparticles (CNPs) at 6 week of age.

Items	CNPs level (mg/kg diet)					SEM	P-Value	
	0	150	300	450	600		Linear	Quadratic
RBCs count (× 10^6^/ μl)	3.30^a^	3.43^a^	3.51^a^	2.76^b^	2.74^b^	0.04	< 0.050	0.49
HGB (g/dl)	18.30^a^	17.50^a^	17.59^a^	14.20^b^	13.61^b^	0.7	< 0.050	0.289
HCT (%)	42.30^a^	43.10^a^	42.51^a^	34.90^b^	33.50^b^	1.64	< 0.050	0.18
WBCs count (× 10^3^/μl)	19.17	19.41	18.91	18.36	18.19	1.15	0.312	0.45
HET (%)	35.00^a^	34.00^a^	33.51^ab^	32.00^b^	32.01^b^	1.06	< 0.050	0.151
LYM (%)	63.00^b^	64.20^b^	64.50^b^	66.00^a^	66.59^a^	1.03	< 0.050	0.286
HET / LYM ratio	0.55^a^	0.53^a^	0.52^a^	0.48^b^	0.48^b^	0.06	< 0.050	0.391
Cortisol (µg/dl)	20.72^a^	19.94^a^	18.53^b^	14.50^c^	13.17^c^	1.05	< 0.050	0.191

^a, b, c^ Mean values in the same row with different superscript letters were significantly. (n = 10).

RBCs, Red blood cells; HGB, Hemoglobin; HCT, Hematocrit; WBCs, White blood cells; HET, Heterophil; LYM, Lymphocyte

### Biochemical indices

As summarized in Table 5, supplementation of CNPs at levels of 300 mg/kg and above significantly reduced serum levels of aspartate aminotransferase (AST), creatinine, urea, and uric acid (P<0.05). CNPs supplementation had no significant effect on serum alanine aminotransferase (ALT) levels (P>0.05). Furthermore, the results indicated that serum HDL concentration was not significantly affected by supplementation with different levels CNPs (P>0.05). However, CNPs at 300 and 450 mg/kg of feed significantly reduced serum levels of cholesterol, triglycerides, and LDL (P<0.05). The highest level of CNPs (600 mg/kg) did not produce a significant effect on serum lipid parameters compared to the control group (P>0.05).

**Table 5:** Serum biochemical metabolites of broiler chicks as affected by dietary supplementation of cinnamon nanoparticles (CNPs) at 6 week of age.

Items	CNPs level (mg/kg diet)					SEM	P-Value	
	0	150	300	450	600		Linear	Quadratic
ALT (U/L)	8.09	7.31	7.01^c^	7	6.1	1.06	0.11	0.421
AST (U/L)	166.44^a^	165.29^a^	129.29^b^	130.00^b^	131.44^b^	5.26	< 0.050	0.19
Creatinine (mg/dl)	0.33^a^	0.34^a^	0.25^b^	0.25^b^	0.26^b^	0.01	< 0.050	0.41
Urea (mg/dl)	4.47	4.15	4.88	3.42	3.42	0.16	0.21	0.45
Uric Acid (mg/dl)	0.063^a^	0.032^b^	0.008^c^	0.019^c^	0.008^c^	0.001	< 0.050	0.52
TC (mg/dl)	162.12^a^	122.32^b^	114.41^bc^	110.80^bc^	101.59^c^	5.21	< 0.050	0.365
TG (mg/dl)	145.89^a^	135.89^ab^	127.14^b^	109.9^bc^	96.95^c^	2.81	< 0.050	0.35
LDL (mg/dl)	37.24^a^	35.39^a^	36.11^a^	33.39^b^	32.81^b^	1.02	< 0.050	0.18
HDL (mg/dl)	11.36	11.09	10.08	10.2	12.08	1.32	0.19	0.21

^a, b, c^ Mean values in the same row with different superscript letters were significantly. (n = 10).

ALT, Alanine transaminase; AST, Aspartate transaminase; TC, Total cholesterol; TG, Triglyceride; LDL, Low density lipoprotein; HDL, High density lipoprotein.

## Discussion

The present study indicates that dietary CNP could be a potential feed additive to enhance broiler chickens' performance and antioxidant capacity. In line with our results, the group supplemented with 450 mg/kg of CNPs showed a notably low feed conversion ratio (FCR = 1.26), which is unusual in standard commercial settings. This value was verified through repeated data checking and accurate feed intake and body weight gain monitoring. We hypothesize that the synergistic effects of enhanced nutrient digestibility, antioxidant activity, and improved gut health contributed to this highly efficient utilization of feed. Consistent with our result, the inclusion of CNPs in broiler chickens' diet (Mohammad et al., 2025) and fish (Abdel-Tawwab et al., 2018) enhanced diet digestion and nutrient digestibility, leading to improved nutrient utilization, which in turn improved growth. This hypothesis was supported by the high activities of digestive enzymes represented herein.

On the other hand, CNPs may inhibit potential pathogens in the digestive tract, may enhance the population of beneficial microorganisms, and/or may enhance microbial enzyme activities that consequently improve feed digestibility and nutrient absorption (Abdel-Tawwab et al., 2018; Mohammad et al., 2025). Moreover, a study by Cross et al. (2007) demonstrated that the phenolic compounds present in essential oils exhibit potent antimicrobial and antioxidant properties and contribute positively to performance enhancement by reducing pathogenic bacteria in the gastrointestinal tract and stimulating the secretion of digestive enzymes.

Several studies have shown that dietary supplementation with cinnamon nanoparticles reduced the population of pathogenic bacteria in the gastrointestinal tract in broiler chickens (Mohammad et al., 2025), fish (Abdel-Tawwab et al., 2018), and rats (Ramezani et al., 2022). The antibacterial effects of cinnamon have also been previously reported (Chang et al., 2001; Tabatabaei et al., 2015; Kallel et al., 2009). In this respect, Toghyani et al. (2011); Baghban Kanan et al. (2016), and Fathi et al. (2017) found that broiler chickens fed diets containing 3, 5, and 2 g/kg cinnamon powder respectively, resulted in significantly greater performance than the other diets.

In the present study, high levels of CNPs (600 mg/kg of feed) resulted in reduced feed intake. This may be attributed to the intense aroma or pungent taste of the bioactive components in unprotected essential oils, which are often present in high concentrations. Excessive inclusion of such compounds may lead to feed refusal and decreased intake in poultry (Hernandez et al., 2004). The optimum CNPs level herein is 450 mg/kg diet; meanwhile, in the aforementioned studies, significantly higher levels of cinnamon were reported to exert beneficial effects. This comparison indicates that cinnamon nanoform is more efficient than its ordinary form. These results may be because the CNPs remained in the bloodstream for a longer period, facilitating its good bioavailability (Abdel-Tawwab et al., 2018; Mohammad et al., 2025). In a similar study, Rahmani et al. (2017, 2018), used curcumin and curcumin nanoparticles in diets for broiler chickens. They found that growth performance, immunity, antioxidant, and antibacterial activity were more efficient with nanoparticles than the traditional form.

Activity of antioxidant enzymes and lipid peroxidation (indicated by MDA) products are indicators of oxidative cell damage and examples of the toxic mechanisms of reactive oxygen species (ROS), which are involved in pathological processes and in the etiology of many broiler chickens diseases (Arab et al., 2006; Fathi et al., 2022). Thus, they are commonly used as biomarkers and quick responses to ROS generation. The present study evoked that dietary CNPs had antioxidant activity where MDA level and activities of SOD and GSH-Px increased significantly in birds fed CNPs-enriched diets. These results may be because cinnamon contains large amounts of bioactive molecules including essential oils (cinnamic aldehyde and cinnamyl aldehyde), polyphenol, tannins, saponins, flavonoids and carbohydrates, which show antioxidant properties (Kwon et al., 2009; Luczaj et al., 2009; Gruenwald et al., 2010; Kallel et al., 2019). Many studies reported that cinnamon has been documented to show antioxidant activity in broiler chickens (Baghban Kanan et al., 2016; Valavi et al., 2016; Shirzadegan and Rezaeipour, 2016; Fathi et al., 2017; Mohammad et al., 2025), fish (Abdel-Tawwab et al., 2018; Ravardshiri et al., 2020), and rat (Ramezani et al., 2022).

The inclusion of cinnamon nanoparticles (CNPs) in the diet led to a reduction in red blood cell count (RBC), hemoglobin (HGB), hematocrit (HCT), heterophils (HET), and cortisol levels, while lymphocyte (LYM) counts increased. Assessing stress in chickens typically involves multiple parameters related to metabolism, immune function, antioxidant responses, and stress biomarkers (Puvadolpirod and Thaxton, 2000). The heterophil-to-lymphocyte (H/L) ratio is a widely accepted indicator of stress in birds; stress generally elevates heterophil counts and lowers lymphocyte levels, increasing the H/L ratio (Siegel, 1995). The observed simultaneous decrease in cortisol and heterophils alongside increased lymphocytes following CNP supplementation suggests potential anti-stress effects.

However, the significant decreases in RBC, HGB, and HCT observed at higher doses of CNPs may indicate an adverse impact on oxygen transport capacity and overall physiological status. While cinnamon nanoparticles possess potent antioxidant properties capable of scavenging free radicals through their metabolites (Huang et al., 2017; Han et al., 2020; Hu et al., 2021), these hematologic changes could reflect complex physiological responses, including modulation of erythropoiesis. Previous studies have suggested that antioxidant compounds might suppress erythropoietin secretion from the kidneys, leading to reduced red blood cell production (Chattopadhyay et al., 2004; Lee et al., 2003).

Therefore, although CNP supplementation shows promising immunomodulatory and anti-stress effects, the potential negative consequences on red blood cell parameters at higher doses warrant careful consideration. Further studies are recommended to clarify the dose-dependent effects and underlying mechanisms of cinnamon nanoparticles in broiler health.

The results of the present study showed that dietary supplementation with CNPs significantly reduced the serum levels of AST, TG, TC, creatinine, uric acid, and LDL in broiler chickens. these reductions should not be interpreted as indicative of poor health in the control group, as the values remained within the physiological range reported for healthy broilers. Rather, these findings demonstrate the favorable modulatory effects of CNPs on hepatic and renal function**s**, likely due to their high antioxidant and anti-inflammatory potential.

Lipid-lowering effects of cinnamon have been well documented in other animals and humans (Rahman et al., 2013; Maierean et al., 2017; Santos and da Silva, 2018). Cinnamon improves lipid-related blood biochemistry by directly affecting lipid metabolism. It has been suggested that antioxidants reduce mevalonate production by inhibiting the activity of HMG-CoA reductase. Plant phenolic compounds can reduce LDL-C oxidation and increase serum HDL-C levels (Weinbrenner et al., 2004; Baker et al., 2008). Furthermore, antioxidants lower triglyceride levels by enhancing their hepatic clearance and reducing hepatic triacylglycerol lipase activity (Ahmadipour et al., 2015). Studies have shown that flavonoids and anthocyanins help prevent chronic cardiovascular disease and atherosclerosis by scavenging free radicals, inhibiting LDL-C oxidation, and reducing blood levels of triglycerides and cholesterol (Ustundag and Ozdogan, 2015).

In the present study, the improved blood biochemistry parameters parallelled the increase in body weight gain and feed efficiency, suggesting enhanced nutrient utilization and physiological homeostasis. When we match the antioxidant activity, liver enzyme profile, and kidney-related metabolites, it can be concluded that cinnamon helped birds to maintain oxidative and metabolic balance, thereby supporting better growth and overall health.

One limitation of the present study is the absence of performance data at days 14 and 28. Including these intermediate measurements could have offered a clearer understanding of growth dynamics and the temporal effects of the supplements. Future studies are encouraged to evaluate performance at multiple time points during the growth cycle to gain more comprehensive and practical insights. While the present study demonstrated the beneficial effects of cinnamon nanoparticles on growth performance, antioxidant status, and selected physiological parameters in broiler chickens, further research is warranted to optimize dosing strategies, evaluate long-term effects, and assess the mechanisms at the molecular level. Future studies should also investigate the interaction of CNPs with other dietary components, their stability during feed processing, and their impact under different environmental or management stressors. Additionally, exploring the effects of CNPs on gut microbiota composition and immune gene expression could offer deeper insights into their mode of action.

## Conclusion

The findings of this study indicate that dietary supplementation with 450 mg/kg of cinnamon nanoparticles (CNPs) positively influenced broiler growth performance by improving feed efficiency and enhancing antioxidant activity, while also reducing mortality rates. Moreover, supplementation at this level showed beneficial effects on liver and kidney function parameters. Hematological indices exhibited a complex response to CNP supplementation; while improvements in immune-related parameters such as increased lymphocyte counts and reduced cortisol and heterophil levels suggest anti-stress effects, reductions in red blood cell counts, hemoglobin, and hematocrit at higher doses highlight the need for cautious interpretation. Therefore, 450 mg/kg appears to be an optimal dose balancing growth performance, antioxidant benefits, and physiological health in broilers. Further research is warranted to fully elucidate the mechanisms behind hematological changes and to confirm the safety of long-term CNP supplementation.

## **Author contributions**

Mokhtar Fathi: Conceptualization, Methodology, Investigation, Data curation, Formal analysis, Project administration, Writing – original draft. Kianoosh Zarrinkavyani: Conceptualization, Methodology, Supervision, Validation, Writing – review & editing. Zahra Biranvand: Methodology, Resources, Technical support. Ali Hashem Mohammad: Methodology, Resources, Technical support.

## Disclosures

We declare that we have no financial and personal relationships with other people or organizations that can inappropriately influence our work, and there is no professional or other personal interest of any nature or kind in any product, service and/or company that could be construed as influencing the content of this paper.
